# A 90-year-old patient presenting with postoperative hypotension and a new murmur: a case report

**DOI:** 10.1186/1752-1947-8-363

**Published:** 2014-11-10

**Authors:** Nicholas L Hartog, Aparna Kamath

**Affiliations:** 1 Department Internal Medicine, University of Iowa Hospital and Clinics, 200 Hawkins Drive, Iowa City, IA 52242, USA

**Keywords:** Postoperative hypotension, Dynamic left ventricular outflow tract obstruction

## Abstract

**Introduction:**

Hospitalists are frequently consulted on postoperative patients with hypotension. Postoperative hypotension is common and can be due to variety of causes. Systolic anterior motion of the mitral valve leading to left ventricular outflow tract obstruction is a rare cause of postoperative hypotension and can occur without prior structural heart disease. A high index of suspicion can lead to early recognition of this unique condition.

**Case presentation:**

A 90-year-old Caucasian woman with no known structural heart abnormality was admitted to the intensive care unit with hypotension after a left hip arthroplasty revision. A transthoracic echocardiogram revealed systolic anterior motion of the mitral valve and dynamic left ventricular outflow tract obstruction as the likely cause of her hypotension. Our patient was treated with fluid resuscitation and phenylephrine with improvement in blood pressure. A repeat echocardiogram on postoperative day 5 showed resolution of the left ventricular outflow tract obstruction. Intraoperative vasodilatation and volume loss that caused underfilling of the left ventricle likely led to dynamic outflow tract obstruction in our patient.

**Conclusions:**

Hospitalists should be aware of systolic anterior motion of the mitral valve as a rare peri-operative complication in patients with or without underlying cardiac pathology as it is treated differently than other causes of peri-operative hypotension. Clinical suspicion, early recognition, and prompt treatment can improve clinical outcomes in these patients.

## Introduction

Systolic anterior motion (SAM) of the mitral valve leading to left ventricular outflow tract (LVOT) obstruction is usually associated with hypertrophic cardiomyopathy [1,2]. Other cardiac pathology, including something as innocuous as hypertension, has been linked as a predisposing factor for developing SAM of the mitral valve (MV). Peri-operative hypotension is one of the most common operative complications with a variety of causes [3]. Here we present the case of a 90-year-old woman with no underlying cardiovascular anomalies who developed SAM of the mitral valve and dynamic LVOT obstruction during a hip arthroplasty revision.

## Case presentation

**Figure 1:**
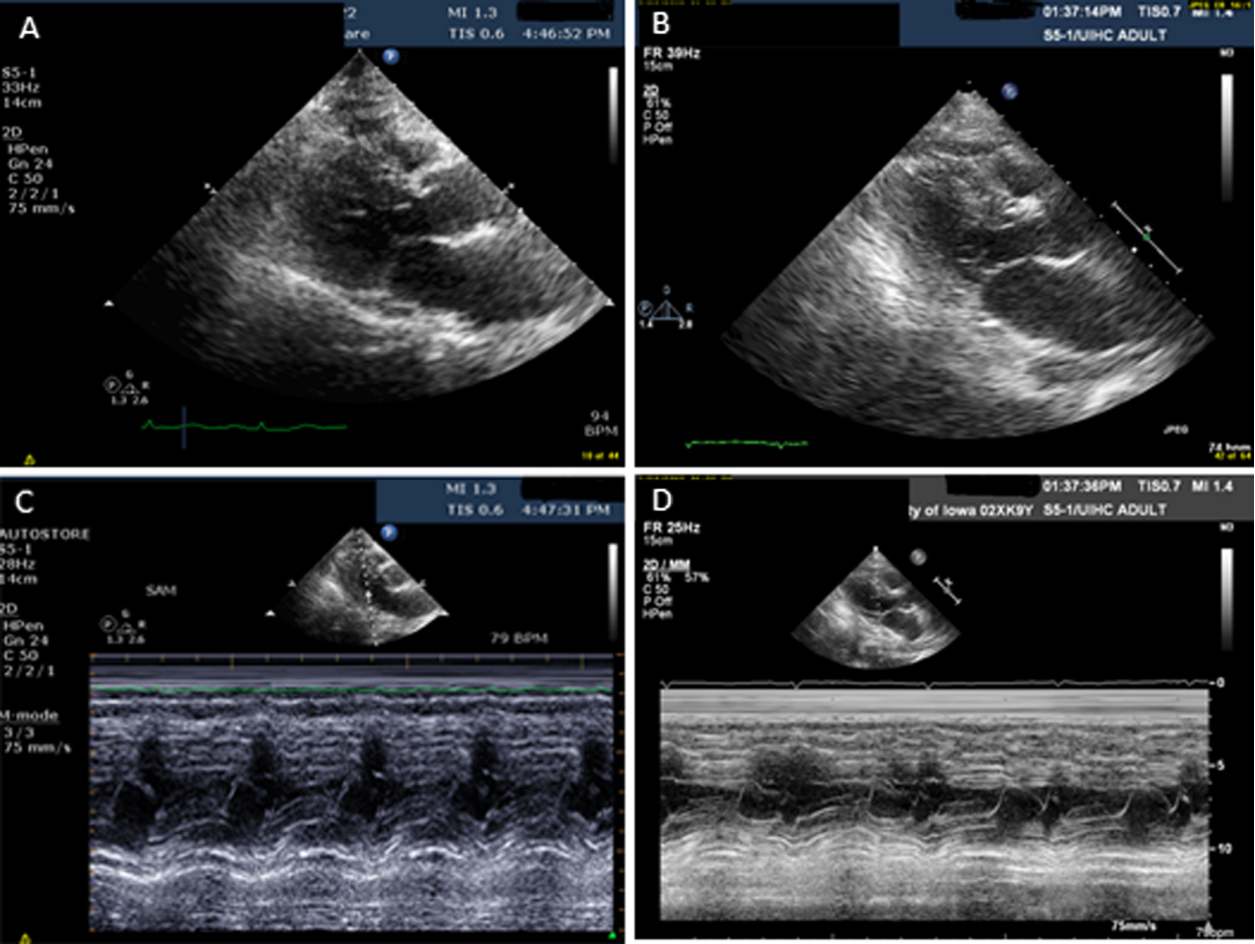
**Parasternal long-axis view during systole and mitral valve M-mode. (A)** Parasternal long-axis view during systole - immediate postoperative period. **(B)** Parasternal long-axis view during systole - postoperative day 5. **(C)** Mitral valve M-mode - immediate postoperative period. **(D)** Mitral valve M-mode - postoperative day 5.

**Figure 2:**
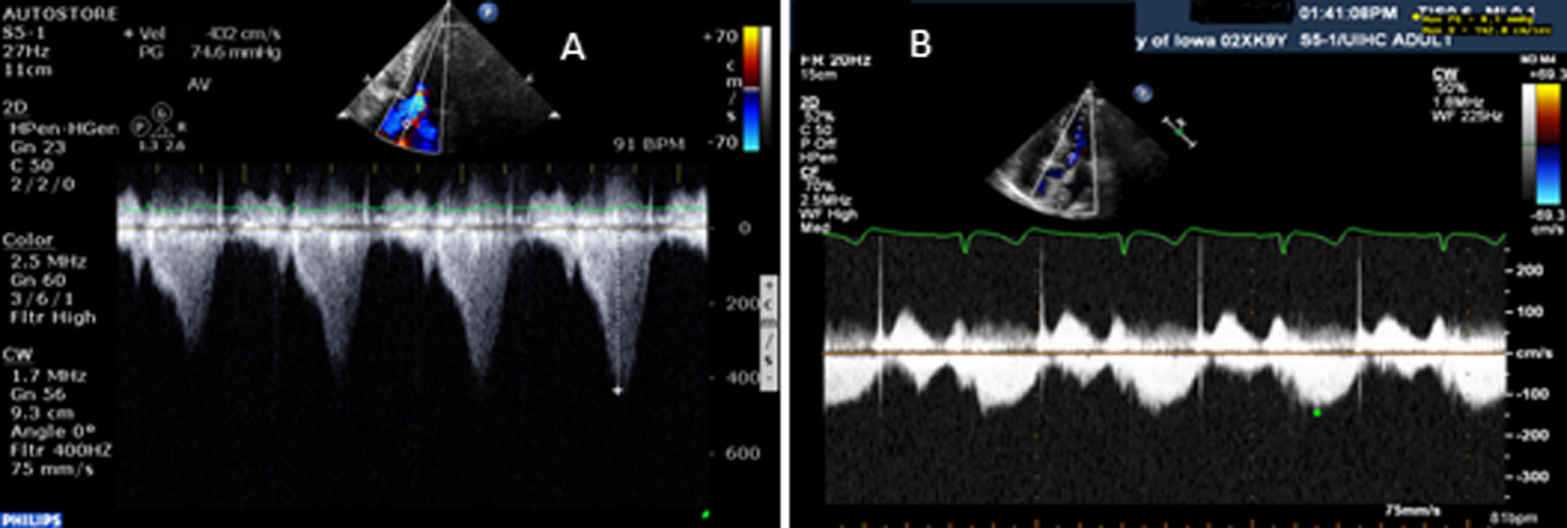
**Continuous-wave Doppler through the left ventricular outflow tract. (A)** Continuous wave Doppler through the left ventricular outflow tract - immediate postoperative period. **(B)** Continuous-wave Doppler through the left ventricular outflow tract - postoperative day 5.

A 90-year-old Caucasian woman with a past medical history significant for provoked upper extremity deep venous thrombosis and *Clostridium difficile* colitis with subsequent ileostomy was admitted to our orthopedic service for a planned left hip arthroplasty revision and correction of acetabular protrusion. Preoperatively our patient denied chest pain or shortness of breath. She was unable to achieve four metabolic equivalents secondary to hip pain. Past surgeries included left hip arthroplasty, bilateral total knee replacement, hysterectomy, appendectomy, and cholecystectomy without any cardiopulmonary complications. A preoperative examination was unremarkable with all vital signs within normal limits and no murmurs, gallops or rubs on cardiac auscultation. Her only medications were omeprazole and vitamin B12. Patient risk was stratified as average risk for an intermediate risk procedure. Intraoperatively our patient was given regional, spinal, and monitored anesthetic care (MAC), which were well tolerated. She initially received midazolam prior to morphine spinal anesthetic. Due to the procedure length, she was converted to general anesthesia using a laryngeal mask airway. Fentanyl, midazolam, and propofol were used to convert to general anesthesia. Shortly after the transition to general anesthesia, she converted from normal sinus rhythm to sinus tachycardia. Unexpected hypotension near the end of operation required 3200cc of crystalloid fluids, 500cc albumin, and two units of packed red blood cells. Postoperatively she was transferred to our surgical intensive care unit.On postoperative evaluation, our patient was alert, comfortable, and without any complaints. Her vital signs revealed a blood pressure of 89/46mmHg and a heart rate of 95 beats per minute on a phenylephrine drip. A physical examination was significant for a new-onset grade 4/6 systolic murmur at the apical area radiating to the axilla. She was breathing comfortably without accessory muscle use and her extremities were warm with 2+ pulses. Her postoperative hemoglobin dropped to 7mg/dL from 12mg/dL preoperatively; her electrocardiogram, renal function, and troponin results were negative. An emergent transthoracic echocardiogram (TTE) revealed dynamic LVOT obstruction with peak velocity of 4.2L/m/s and moderate to severe mitral regurgitation due to SAM of the anterior MV leaflet (Figure 1). Her LV outflow gradient was 65 to 70mmHg (normal <30mmHg). By the end of postoperative day 1, our patient was weaned off the phenylephrine. On postoperative day 2, she no longer had a cardiac murmur on auscultation. A follow-up echocardiogram three days later revealed resolution of outflow obstruction and resolution of mitral regurgitation (Figure 1 and Figure 2).

## Discussion

Hypotension is a common problem in the postoperative period with many potential etiologies. Causes fall into three main categories: hypovolemia, distributive, and cardiogenic [4]. SAM of the MV is an important cause of postoperative hypotension. Although most commonly associated with structural abnormalities, SAM of the MV has been rarely reported in patients without known cardiac pathology after undergoing general anesthesia [5]. Luckner *et al.* reported three cases of patients with no cardiac history that developed SAM of the MV in the peri-operative period. Similar to cases reported by Chockalingam *et al.* our patient had no evidence of underlying structural abnormalities and a follow up TTE showed resolution of SAM of the MV [6,7].

SAM of the MV can cause severe postoperative hypotension and should be considered in patients with a new systolic murmur. Hypovolemia due to blood loss or vasodilator effect of anesthetic agents can result in left ventricle (LV) underfilling [1]. This reduces the LVOT size thus resulting in a hyperdynamic LV. Underfilling of the LV also changes geometry of the ventricle moving the papillary muscles relatively anterior and inwards [1]. The hyperdynamic state raises outflow tract velocity, increasing drag forces on the MV resulting in LVOT obstruction [1]. Without prompt treatment, dynamic LVOT obstructions are associated with up to 20% risk of sudden death [1].

## Conclusions

While the majority of patients who develop SAM of the MV and dynamic LVOT obstruction have underlying structural abnormalities or previous cardiovascular pathology, we present a case where the patient had neither. SAM of the MV valve can be promptly identified with a TTE. Because patients with a dynamic LVOT obstruction secondary to SAM of the MV are preload dependent, proper management includes volume resuscitation, peripheral vasoconstriction, and beta-adrenoceptor blockade [1,2]. Prompt identification and appropriate treatment is essential for patient survival with this acute illness.

## Consent

Written consent was obtained from the patient for publication of this case report and any accompanying images. A copy of the written consent is available for review by the Editor-in-Chief of this journal.

## Abbreviations

LVOT: Left ventricular outflow tract; MV: Mitral valve; SAM: Systolic anterior motion; TTE: Transthoracic echocardiogram.

## Competing interests

The authors of this manuscript declare that neither have any competing interests.

## Authors’ contributions

NH and AK were both responsible for originally taking care of this patient. NH was responsible for writing the manuscript. AK was also a major contributor in the writing of the manuscript. Both authors read and approved the final manuscript.
